# Estimation of Obesity Levels through the Proposed Predictive Approach Based on Physical Activity and Nutritional Habits

**DOI:** 10.3390/diagnostics13182949

**Published:** 2023-09-14

**Authors:** Harika Gozde Gozukara Bag, Fatma Hilal Yagin, Yasin Gormez, Pablo Prieto González, Cemil Colak, Mehmet Gülü, Georgian Badicu, Luca Paolo Ardigò

**Affiliations:** 1 Department of Biostatistics and Medical Informatics, Faculty of Medicine, Inonu University, Malatya 44280, Turkey; harika.gozukara@inonu.edu.tr; 2 Department of Management Information Systems, Faculty of Economics and Administrative Sciences, Sivas Cumhuriyet University, Sivas 58140, Turkey; yasingormez@cumhuriyet.edu.tr; 3 Sport Sciences and Diagnostics Research Group, GSD-HPE Department, Prince Sultan University, Riyadh 11586, Saudi Arabia; pprieto@psu.edu.sa; 4 Department of Sports Management, Faculty of Sport Sciences, Kirikkale University, Kirikkale 71450, Turkey; mehmetgulu@kku.edu.tr; 5 Department of Physical Education and Special Motricity, Transilvania University of Brasov, 00152 Brasov, Romania; georgian.badicu@unitbv.ro; 6 Department of Teacher Education, NLA University College, Linstows Gate 3, 0166 Oslo, Norway; luca.ardigo@nla.no

**Keywords:** obesity, machine learning, physical activity, nutritional habits, classification

## Abstract

Obesity is the excessive accumulation of adipose tissue in the body that leads to health risks. The study aimed to classify obesity levels using a tree-based machine-learning approach considering physical activity and nutritional habits. Methods: The current study employed an observational design, collecting data from a public dataset via a web-based survey to assess eating habits and physical activity levels. The data included gender, age, height, weight, family history of being overweight, dietary patterns, physical activity frequency, and more. Data preprocessing involved addressing class imbalance using Synthetic Minority Over-sampling TEchnique-Nominal Continuous (SMOTE-NC) and feature selection using Recursive Feature Elimination (RFE). Three classification algorithms (logistic regression (LR), random forest (RF), and Extreme Gradient Boosting (XGBoost)) were used for obesity level prediction, and Bayesian optimization was employed for hyperparameter tuning. The performance of different models was evaluated using metrics such as accuracy, recall, precision, F1-score, area under the curve (AUC), and precision–recall curve. The LR model showed the best performance across most metrics, followed by RF and XGBoost. Feature selection improved the performance of LR and RF models, while XGBoost’s performance was mixed. The study contributes to the understanding of obesity classification using machine-learning techniques based on physical activity and nutritional habits. The LR model demonstrated the most robust performance, and feature selection was shown to enhance model efficiency. The findings underscore the importance of considering both physical activity and nutritional habits in addressing the obesity epidemic.

## 1. Introduction

The obesity burden has increased worldwide in recent decades [1]. According to the World Health Organization (WHO), obesity is defined as excessive obesity and abnormal accumulation of adipose tissue in the human body that implies health risks. Individuals with a body mass index (BMI) greater than 30 are considered obese, whereas when the BMI is between 25 and 30, they are considered overweight [2]. Obesity leads to numerous problems in different fields (health, demographics, labor, family, and economics).

From a health point of view, obesity increases the risk of chronic diseases, cardiovascular disease, various types of cancer, musculoskeletal disorders, metabolic syndrome, diabetes mellitus (due to increased insulin resistance), and kidney disease. It also increases inflammatory processes and produces adverse vascular changes such as arterial stiffness [3,4].

Economically, for example, in 2019, in terms of per capita income, obesity accounted for USD 17 in India and USD 940 in Australia. The economic costs of obesity represented 0.8% of India’s gross domestic product (GDP) and 2.4% of Saudi Arabia’s. Furthermore, the expense contributes to 1.52% of Peruvian GDP spending and 1.83% of Mexican GDP expenditure [5]. By 2060, if preventive measures are not taken, the economic impact of obesity could reach, on average, 3.6% of the gross domestic product in all countries [6].

All these data reveal the magnitude of the obesity problem. In fact, since 1997, the WHO considered obesity a global epidemic and a major public health problem [7,8]. To reverse this situation, public authorities, private institutions and companies, and international organizations promote prevention and treatment campaigns focused on physical activity and nutritional habits [9], since both are the two main risk factors in preventing non-communicable diseases [10].

On the one hand, nutrition significantly impacts health and the possibility of developing obesity [11]. In this sense, it is important to remember that the macronutrients and micronutrients present in food play critical roles in humans, including energetic, structural, absorption, insulation, protection, and transport functions. They are also essential for tissue growth and repair and act as activators and regulators of the chemical reactions that occur in the human body [12]. Thus, it is well known that an excessive intake of calories can lead to obesity [13]. However, energy balance may not be the only reason for obesity [14]. Similarly, unhealthy and unbalanced diets and low-quality foods can alter the intestinal microbiota and, thus, increase the risk of suffering from obesity [15]. Moreover, it should be considered that the level of exposure of human beings to a diet is maximal, since all individuals ingest food daily, all or most of the days of their lives [16]. Thus, the diet’s influence on health and the possibility of suffering from obesity is very high.

On the other hand, according to the current evidence, physical activity is also a key factor in preventing and treating obesity [17]. The positive effect of physical activity has been observed in subjects of all ages, from childhood to old age [18,19,20]. However, it must be considered that the frequency, intensity, duration, and type of exercise can reduce obesity. Thus, aerobic exercise of moderate to intense intensity with a weekly frequency of three to five times per week favors weight loss due to increased caloric expenditure [17]. The benefits are more significant when aerobic exercise is combined with a diet [17]. Strength training also improves the reduction of obesity levels [21]. These improvements have usually been attributed to increased basal metabolism [21]. However, it has also been documented that muscle tissue may release extracellular vesicles that promote lipolysis [21]. In this context, to improve obesity prevention strategies, the scientific community and health professionals are currently registering and analyzing extensive datasets to gain in-depth knowledge and understanding of this problem and thus diagnose, prevent, monitor, and cure obesity more effectively. Hence, the current research paper intends to classify obesity levels through a proposed tree-based machine-learning approach based on physical activity and nutritional habits.

## 2. Materials and Methods

### 2.1. Study Design, Ethical Approval, and Data Features

The current study employed an observational design that collected data from a public dataset via a web page that used a poll to assess participants’ eating habits and various attributes to determine their physical condition. The Inonu University Health Sciences Non-Interventional Clinical Research Ethics Committee approved this study (approval number: 2023/4677). The data for this study included the eating habits and physical activity levels of 498 participants aged between 14 and 61 years, which were used to estimate their obesity levels [22]. A survey was administered through a web-based platform where respondents anonymously answered each question in the related study. The relevant paper provides data about the dietary patterns and physical status of individuals from Colombia, Peru, and Mexico. The public dataset in this study has 17 variables, explained below:

- Gender: categorical variable that shows the biological sex of the individual (male or female).
- Age: numerical variable that shows the individual’s age in years.
- Height: numerical variable that shows the individuals’ height in meters.
- Weight: numerical variable that shows the individuals’ weight in kilograms.
- Family history of overweight: categorical variable that shows if the individual has a family member who is overweight or obese (yes or no).
- Frequently consumed high-calorie food (FAVC): categorical variable that shows if the individual often eats high-calorie food (yes or no).
- Frequency of consumption of vegetables (FCVC): ordinal variable that shows how often the individual eats vegetables (1 = never, 2 = sometimes, 3 = always).
- Number of main meals (NCP): ordinal variable that shows how many main meals the individual has daily (1 = between 1 and 2, 2 = three, 3 = more than three, 4 = no answer).
- Consumption of food between meals (CAEC): ordinal variable that shows how often the individual eats food between meals (1 = no, 2 = sometimes, 3 = frequently, 4 = always).
- SMOKE: categorical variable that shows whether the individual smokes or not (yes or no).
- Consumption of water daily (CH2O): ordinal variable that shows how much water the individual drinks daily (1 = less than a liter, 2 = between 1 and 2 L, 3 = more than 2 L).
- Monitor calorie intake (SCC): categorical variable that shows if the individual keeps track of their caloric intake (yes or no).
- Frequency of physical activity (FAF): ordinal variable that shows how often the individual does physical activity (1 = never, 2 = once or twice a week, 3 = two or three times a week, 4 = four or five times a week).
- Time using electronic devices (TUE): ordinal variable that shows how long the individual uses electronic devices (0 = none, 1 = less than an hour, 2 = between one and three hours, 3 = more than three hours).
- Consumption of alcohol (CALC): ordinal variable that shows how often the individual drinks alcohol (1 = no, 2 = sometimes, 3 = frequently, 4 = always).
- Type of transportation used (MTRANS): categorical variable that shows what kind of transportation the individual uses (automobile, motorbike, bike, public transportation, walking).
- Level of obesity according to body mass index (NObesity): ordinal variable that shows the obesity level of the individual according to their BMI (insufficient weight normal weight, overweight level I, overweight level II, obesity type I, obesity type II, obesity type III). The related attribute was the primary outcome [22].

After performing all the calculations to compute the BMI of each participant, the WHO criteria were applied to classify the obesity levels as follows: underweight = BMI less than 18.5; normal = BMI between 18.5 and 24.9; overweight = BMI between 25.0 and 29.9; obesity I = BMI between 30.0 and 34.9; obesity II = BMI between 35.0 and 39.9; and obesity III = BMI higher than 40. The WHO criteria are based on the relationship between BMI and the risk of chronic diseases and mortality. Table 1 presents the class imbalance distribution of obesity levels in the original dataset.

### 2.2. Data Preprocessing

There were 498 participants in the original dataset. However, when the obesity levels of the participants were examined, it was determined that there was a very high level of class imbalance. Due to the biased results of ML algorithms in datasets with class imbalance problems, it was attempted to eliminate the imbalance between classes (obesity levels). As a result, a dataset with 2009 samples and 16 features adjusted with the Synthetic Minority Over-sampling Technique-Nominal Continuous (SMOTE-NC) approach was used to predict obesity levels. Thanks to the SMOTE-NC approach, the class imbalance problem in the data set was resolved. SMOTE-NC is an extension of the original SMOTE algorithm designed to handle datasets with both nominal and continuous features. SMOTE-NC was developed to address the imbalance problem in classification tasks where nominal and continuous attributes characterize the minority class. The SMOTE-NC algorithm generates synthetic samples for the minority class by interpolating between feature vectors of neighboring instances. It extends the original SMOTE algorithm by handling both nominal and continuous features in a unified manner [23]. SMOTE-NC handles nominal attributes differently than continuous attributes and keeps the original labels of categorical features in the resampled data. As a result of SMOTE-NC, a total of 2009 observations were generated, 287 for each obesity sublevel based on the available data.

Feature selection was performed to identify the most important obesity-related features that could contribute to the estimation of obesity level accurately. For this purpose, the Recursive Feature Elimination (RFE) technique was used. RFE is a feature selection technique commonly used in machine learning and data analysis. Its purpose is to select the most relevant features from a given dataset, aiming to improve the model’s performance by reducing the dimensionality of the feature space. RFE iteratively eliminates features with the least importance based on the importance scores assigned by the estimator. This process continues until the desired number of features is reached, or a stopping criterion is met (e.g., a minimum performance threshold is achieved). The main advantage of RFE is that it considers the interactions among features, allowing for selecting of feature subsets that collectively contribute to better predictive performance. However, it can be computationally expensive for large datasets since it requires training the estimator multiple times [24,25]. RFE is a flexible technique that can be applied with various machine-learning algorithms and has proven effective in reducing overfitting, improving model interpretability, and enhancing prediction accuracy by focusing on the most informative features [26].

### 2.3. Data-Generated Training, Testing, and Validation Procedures

While constructing the training, testing, and optimization dataset, equal samples were selected from each class. In this context, 25% of the original dataset for each class was randomly chosen to form a testing dataset, and the remainder were utilized to build a training dataset. As a consequence of this procedure, a testing set with 497 samples, which included 71 samples for each class, and a training set with 1512 samples, which included 216 samples for each class, were developed. After that, two portions were formed from the training dataset for hyperparameter optimization and feature selection processes. For this reason, 20% of the training dataset for each class was randomly picked to construct the testing set for optimization (testingForVal), and reaming was utilized to generate the training set for optimization (trainingForVal). As a consequence of this procedure, a testingForVal set with 301 samples, which contained 43 samples for each class, and a trainingForVal set with 1211 samples, which included 173 samples for each class, were created through the applicable function. This study employed the trainingForVal and testingForVal datasets for hyperparameter optimization and the feature-selection method. Other than that, the training and testing datasets were utilized to train and test the final model that employed the optimal hyperparameters and chosen features.

### 2.4. Model Development

In this study, three classification algorithms, logistic regression (LR), random forest (RF), and Extreme Gradient Boosting (XGBoost), were used to predict obesity levels. These methods were chosen as classifiers in our study because they are easy to apply, have a high accuracy rate in many problems, and can be applied quickly.

**LR:** LR is a data analysis technique that uses mathematics to find relationships between two data factors. This method is a frequently used machine-learning method as it gives high accuracy rates and is fast. It is similar to a linear regression model but is suitable for models in which the dependent variable is a categorical feature. It models the probability that a given input belongs to a particular class. Logistic regression can be extended to handle multiple classes through techniques like one-vs-all (OvA) or softmax regression. It works well when the relationship between input features and the target variable is approximately linear and when there is a need for interpretable results [27,28,29].

**RF:** RF is a popular machine-learning algorithm that belongs to the ensemble learning family. It is primarily used for both classification and regression tasks and is known for its high accuracy, robustness, and ability to handle complex datasets. It is robust to outliers and missing values [30].

**XGBoost:** XGBoost is a powerful learning algorithm used in the field of machine learning. It is mainly used to solve various learning tasks such as regression and classification. XGBoost is an algorithm that provides high performance especially in structured data (such as table data). XGBoost is the high-performance version of the gradient boosting algorithm optimized with various tweaks. The most important features of the algorithm, which Chen and Guestring first proposed, are its ability to achieve a high accuracy rate, prevent over-fitting, manage empty data, and be quick [31].

### 2.5. Hyperparameter Optimization

Similarly to many machine-learning methods, hyperparameters significantly affect the performance of the models used in this study. Because of this important reason, the hyperparameters of the machine-learning models were optimized using Bayesian optimization techniques. It helps find the best combination of hyperparameters for a machine-learning model efficiently by minimizing the number of evaluations of the objective function (model performance) while accounting for uncertainty and noise. Bayesian optimization was chosen because it is more effective and faster than other techniques [32,33]. This technique was implemented using the skopt library in Python [34]. In this library, maximum and minimum values are determined for each hyperparameter space. The values in this range are then optimized using the Gaussian process.

### 2.6. Performance Evaluation Metrics

Accuracy, recall, precision, F1-score, AUC, and precision–recall curve evaluation metrics were used to evaluate and compare the performance of the ML models in the obesity level estimation task. Calculations for performance evaluation metrics were done using the scikit metrics library in Python [35].

**Accuracy:** Accuracy measures the proportion of correct predictions from the total number of predictions. It is calculated as the number of correct predictions divided by the total number of predictions. Accuracy is often used in classification tasks when the classes are balanced. However, it may not be an appropriate metric when dealing with imbalanced datasets [36].

**Precision:** Precision is a metric that quantifies the accuracy of positive predictions. It measures the proportion of correctly predicted positive instances out of the total number of positive predictions. Precision is calculated as the number of true positives divided by the sum of true positives and false positives. Precision is useful when focusing on minimizing false positives [37].

**F1-score:** The F1-score is the harmonic mean of precision and recall. It provides a single metric that combines both precision and recall, which are often inversely related. F1-score is calculated as 2 × (precision × recall)/(precision + recall). F1-score is useful to balance precision and recall in evaluation [38].

**AUC:** The ROC curve (receiver operating characteristic curve) is a graphical representation of a model’s performance, showing the trade-off between its sensitivity (true positive rate) and specificity (true negative rate) across different threshold values. The AUC is a numerical measure derived from the ROC curve, specifically the area under the ROC curve. It quantifies the overall ability of the model to discriminate between the two classes, with higher AUC values indicating better performance [39,40].

**Recall:** Recall is an important metric used in classification to evaluate the performance of a model, especially where the identification of true positive cases is critical. Recall is defined as the ratio of true positive predictions to the total number of actual positive cases (true positives plus false negatives). It measures the model’s ability to correctly identify all positive instances out of all the actual positive instances in the dataset [41,42].

### 2.7. Biostatistical Data and Power Analyses

The data were expressed as frequency (percentage) for overall variables. Qualitative data were analyzed with Pearson’s chi-square test. *p*-values <0.05 were considered as significant. IBM SPSS Statistics version 28.0 for Windows (New York, NY, USA) was used for statistical analyses. A post hoc power analysis revealed 0.9997 power considering an effect size of 0.09, type I error of 0.05, total sample size of 498, and two-tailed alternative hypothesis using G*Power 3.1.9.7 version.

## 3. Results

### 3.1. Biostatistical Results

Table 2 provides descriptive statistics for the data according to obesity levels. Significant associations were found between obesity levels and gender, family history of overweight, FAVC, FCVC, NCP, CAEC, SMOKE, CH2O, SCC, FAF, TUE, CALC, and MTRANS categories (Table 2).

### 3.2. Modeling Results Using All Features for Obesity Level Estimation

Hyperparameter spaces, optimal values of each hyperparameter, hyperparameter space types, and optimal validation accuracy are shown in Table 3. In addition to these values, the gp_minimize function of this library was used with acq_func = ‘EI’ and n_cals = 100.

During the hyperparameter optimization phase, the models were trained using the trainingForVal dataset, and the hyperparameters that had the best accuracy on the testingForVal dataset were selected. Validation accuracy in Table 4 shows the best accuracy on the testingForVal dataset. After the hyperparameter optimization phase, LR, RF, and XGBoost models were trained using a training dataset with the optimal hyperparameters. After the training phase, accuracy, precision, F1-score, AUC, and recall, which are shown in Table 4, were computed on the testing dataset. According to these results, the LR model obtained the best performance measures for all metrics.

### 3.3. Modeling Results with the Biomarker Candidate Selected Features for Obesity Level Estimation

The RFE technique was used with LR, RF, and XGBoost classifiers separately to measure the effect of each feature in predicting obesity level. For this purpose, in this method, the worst-performing attributes are eliminated step by step, starting from the entire attribute set until the best attribute subset is found [24]. In the first step of this phase, in addition to the hyperparameters shown in Table 3, the number of features was also optimized using the Bayesian optimization technique for each classification method. For this purpose, the *gp_minimize* function of the skopt library was used with *acq_func = ‘EI’* and *n_cals = 100*. In each call, a model trained using a selected feature on the trainingForVal dataset, and the hyperparameters and feature set that had the best accuracy on the testingForVal dataset were determined. Hyperparameter spaces, optimal values of each hyperparameter, hyperparameters space types, optimal validation accuracy, and selected features are shown in Table 5.

According to validation accuracy results shown in Table 5, LR and RF obtained better accuracy than the full feature, but XGBoost obtained worse accuracy than the full feature. After the hyperparameter optimization phase, LR, RF, and XGBoost models were trained using a training dataset with the optimal hyperparameters and selected features. After the training phase, accuracy, precision, F1-score, AUC, and recall (shown in Table 6) were computed on the testing dataset to observe the effect of feature selection on the test data.

The feature selection process was performed using a wrapper approach that evaluated the performance of different subsets of features on the validation dataset. The validation accuracy results shown in Table 4 indicate that LR and RF achieved better accuracy than the full feature set, while XGBoost performed worse than the full feature set. This suggests that some features may have been redundant or irrelevant for the XGBoost model, while LR and RF could benefit from a reduced feature space. After the hyperparameter optimization phase, LR, RF, and XGBoost models were trained using the training dataset with the optimal hyperparameters and the selected features. The trained models were then tested on the testing dataset to measure their generalization ability. The evaluation metrics used were accuracy, precision, F1-score, AUC, and recall, which are shown in Table 6. The results demonstrated that feature selection improved the performance of LR and RF on all metrics, while XGBoost showed a slight improvement in accuracy, precision, AUC, and recall but a deterioration in F1-score. These findings indicate that feature selection could enhance the efficiency and effectiveness of some machine-learning models, but also introduced some trade-offs or limitations for others.

**Figure 1:**
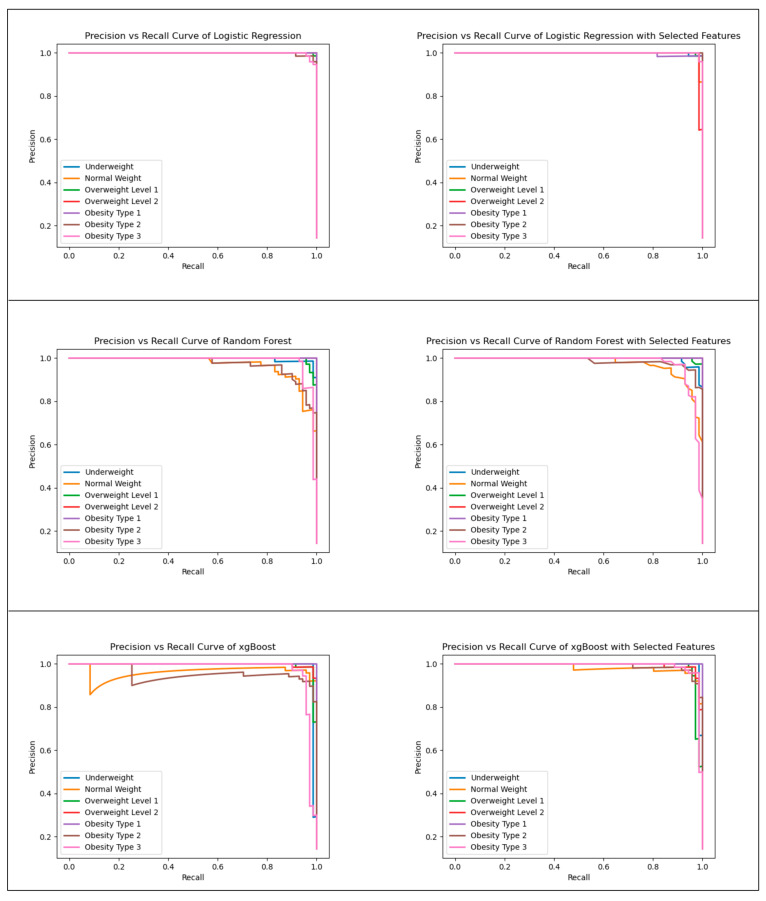
Precision–recall curves of all models.

Based on the analysis outcomes, the LR model demonstrated superior performance across all evaluation metrics, similar to the model trained with the complete set of features. Additionally, the RF model exhibited improved results when employing the selected subset of features, surpassing the performance achieved using the entire feature set for all assessed metrics. Notably, the LR model yielded enhanced outcomes when utilizing the selected features compared to the full feature set, particularly in accuracy, F1-score, AUC, and recall. Conversely, the XGBoost model improved precision results when employing the selected features instead of the full feature set. Considering these comprehensive findings, it can be inferred that training a model using the selected features holds greater significance due to the attainment of superior results while minimizing the complexity of the model. To further elucidate the impact of feature selection, precision–recall curves for each model are illustrated in Figure 1. This visualization effectively highlights the contrast between the outcomes achieved through feature selection and those obtained from the entire feature set, particularly when analyzed on a class-specific basis.

## 4. Discussion

Obesity prevention strategies require comprehensive and accurate data to inform the scientific and health communities about the causes, consequences, and solutions of this condition. Therefore, this study aimed to apply a tree-based machine-learning method to classify obesity levels based on physical activity and nutritional habits. This method can help identify the most relevant factors and patterns associated with obesity, such as the type, frequency, duration, and intensity of physical activity, and the quantity, quality, variety, and timing of food intake. Hence, this method can guide diagnosis, prevention, monitoring, and treatment of this condition, and suggest personalized interventions and recommendations for different obesity levels. For example, this method can help determine the optimal amount and type of physical activity for each obesity level based on the individual’s age, gender, health status, and preferences. Similarly, this method can help design a balanced and nutritious diet plan for each obesity level, considering the individual’s allergies, intolerances, and cultural factors. Moreover, this method can help monitor the progress and outcomes of the interventions and recommendations by tracking changes in obesity levels over time and evaluating their impact on health indicators such as blood pressure, cholesterol, glucose, and inflammation. Furthermore, this method can help treat obesity-related complications and comorbidities, such as diabetes, cardiovascular disease, and depression, by adjusting the interventions and recommendations according to an individual’s needs and responses [43].

This study aimed to compare the performance of different machine-learning models in predicting the outcome of a specific task, using either full features or selected feature sets. The selected features were obtained by applying a feature-selection method that ranked them according to their importance. The feature-selection process was performed using a wrapper approach that evaluated the performance of different subsets of features on the validation dataset. The validation accuracy results indicated that LR and RF achieved better accuracy than the full feature set, while XGBoost performed worse than the full feature set. This result suggests that some features may have been redundant or irrelevant for the XGBoost model, while LR and RF could benefit from reduced feature space. After the hyperparameter optimization phase, LR, RF, and XGBoost models were trained using the training dataset with the optimal hyperparameters and the selected features. The trained models were then tested on the testing dataset to measure their generalization ability. The achieved results demonstrated that feature selection improved the performance of LR and RF on all metrics, while XGBoost showed a slight improvement in accuracy, precision, recall, and AUC but a deterioration in F1-score. These findings indicate that feature selection can enhance the efficiency and effectiveness of some machine-learning models but may also introduce some trade-offs or limitations for others.

The performance metrics used to evaluate the models were accuracy, precision, recall, F1-score, and AUC. According to these results, the LR model obtained the best performance measures for all metrics, similar to those of the model trained using full features. In addition, RF obtained better results using selected features than full features for all metrics. XGBoost obtained better results using the selected features than full features for precision, recall, and AUC. Considering all these results, it was concluded that training a model using the selected features was more meaningful because better results were achieved with less complexity. To further illustrate the effect of feature selection on the performance of the models, precision–recall curves for each model are shown in Figure 1. These curves plot the precision and recall values for different thresholds of the predicted probabilities. Higher precision means that the model is more accurate in predicting positive outcomes, while higher recall means that the model is more sensitive in detecting positive outcomes. As can be seen from Figure 1, the models trained with selected features generally had higher precision and recall values than the models trained with full features, especially for LR and RF. This indicates that feature selection helped reduce the noise and redundancy in the data and improved the generalization ability of the models.

A related study developed a hybrid model that combines three machine-learning techniques: gradient boosting classifier, extreme gradient boosting, and multilayer perceptron. They tested seven different machine-learning algorithms on public datasets from the UCI machine-learning repository and compared their accuracy levels. The hybrid model they proposed could predict and classify obesity with an accuracy of 97.16%, which is higher than that of the individual models and other hybrid models [44]. Another similar article presented three machine-learning methods to forecast obesity in children at age five using real data. The methods used different data sets depending on the available information: (1) one well-child visit, (2) several well-child visits before age two, and (3) several random well-child visits before age five. The models could classify a child’s obesity status (normal, overweight, or obese) at age five with 89%, 77%, and 89% accuracy, respectively [45]. Another study used AI and machine learning to analyze a dataset of EHRs with data on people’s health and lifestyle. They compared three methods, XGB, SVM, and ANN, in classifying people into different obesity categories and determined that XGB was the best method, with very high accuracy rates in their experiments [46]. A systematic literature review selected 93 papers as primary studies from more than 700 papers that addressed obesity issues and identified the key factors that affect and cause obesity in adults. Finally, it explored the machine learning techniques that can be applied to obesity prediction [47]. Within the current study, the LR model emerged as a standout performer in forecasting obesity levels. Compared with those of the RF and XGBoost algorithms, the LR model exhibited superior predictions, fortified by optimal hyperparameters and feature selection. Notably, the LR model’s classification results surpassed those of the models employed in other analogous studies [44,45,46]. These findings underscore the LR model’s efficacy in the context of obesity classification and prediction.

## 5. Conclusions

Within the scope of this study, the LR model emerged as the frontrunner, highlighting a level of performance better than that of alternative methodologies. The strategic integration of feature-selection methods further amplified the model’s efficiency, reinforcing its applicability in real-world scenarios. These findings serve as a poignant reminder of the interconnectedness between physical activity and nutritional habits in combatting obesity.

## Figures and Tables

**Table 1:** Class imbalance distribution of obesity levels in the original dataset.

Obesity Levels	*n*	%
Underweight	34	6.8
Normal Weight	287	57.6
Overweight Level I	47	9.4
Overweight Level II	11	2.2
Obesity Type I	3	0.6
Obesity Type II	58	11.6
Obesity Type III	58	11.6
Total	498	100

**Table 2:** Descriptive statistics for the data according to obesity levels.

Variable	Category	Obesity Levels							*p*-Value
		Underweight	Normal Weight	Overweight Level I	Overweight Level II	Obesity Type I	Obesity Type II	Obesity Type III	
		*n* = 287	*n* = 287	*n* = 287	*n* = 287	*n* = 287	*n* = 287	*n* = 287	
		*n* (%)	*n* (%)	*n* (%)	*n* (%)	*n* (%)	*n* (%)	*n* (%)	
Gender	Female	142 ^a^ (16.70)	141 ^a^ (16.50)	119 ^a,d^ (14.00)	32 ^b^ (3.80)	195 ^c^ (22.90)	131 ^a^ (15.40)	92 ^d^ (10.80)	<0.001
	Male	145 ^a^ (12.50)	146 ^a^ (12.60)	168 ^a,d^ (14.50)	255 ^b^ (22.00)	92 ^c^ (8.00)	156 ^a^ (13.50)	195 ^d^ (16.90)	
Family history of overweight	Yes	167 ^a^ (31.60)	132 ^a,c^ (25.00)	29 ^b^ (5.50)	13 ^b^ (2.50)	0 (0.00)	113 ^c^ (21.40)	75 ^d^ (14.20)	<0.001
	No	120 ^a^ (8.10)	155 ^a,c^ (10.50)	258 ^b^ (17.40)	274 ^b^ (18.50)	2871 (19.40)	174 ^c^ (11.80)	212 ^d^ (14.30)	
FAVC	Yes	106 ^a,c^ (16.40)	79 ^a^ (12.20)	47 ^b^ (7.30)	114 ^c^ (17.70)	100 ^a,c^ (15.50)	91 ^a,c^ (14.10)	108 ^a,c^ (16.70)	<0.001
	No	181 ^a.c^ (13.30)	208 ^a^ (15.20)	240 ^b^ (17.60)	173 ^c^ (12.70)	187 ^a,c^ (13.70)	196 ^a,c^ (14.40)	179 ^a,c^ (13.10)	
FCVC	Newer	30 ^a,d,e,f^ (20.80)	18 ^a,b^ (12.50)	17 ^a,c^ (11.80)	49 ^d^ (34.00)	0 ^1^ (0.00)	16 ^b,c,e^ (11.10)	14 ^b,c,f^ (9.70)	<0.001
	Sometimes	105 ^a^ (11.60)	155 ^b^ (17.20)	191 ^c^ (21.20)	89 ^a^ (9.90)	0 ^1^(0.00)	177 ^b,c^ (19.60)	185 ^b,c^ (20.50)	
	Always	152 ^a^ (15.80)	114 ^b,d,e^ (11.80)	79 ^c^ (8.20)	149 ^a,b^ (15.50)	287 ^1^ (29.80)	94 ^c,d^ (9.80)	88 ^c,e^ (9.10)	
NCP	Between 1 and 2	42 ^a^ (10.60)	52 ^a,b^ (13.10)	77 ^b,c^ (19.40)	58 ^a,b,c^ (14.60)	0 ^1^ (0.00)	88 ^c^ (22.20)	79 ^b,c,d^ (19.90)	<0.001
	Three	169 ^a^ (11.40)	206 ^b,c^ (13.90)	210 ^b,c^ (14.20)	220 ^b^ (14.90)	287 ^1^ (19.40)	186 ^a,c^ (12.60)	201 ^a,b^ (13.60)	
	More than three	76 ^a^ (56.70)	29 ^b^ (21.60)	0 ^1^ (0.00)	9 ^c^ (6.70)	0 ^1^ (0.00)	13 ^b,c^ (9.70)	7 ^c^ (5.20)	
CAEC	No	8 ^a^ (4.90)	35 ^b,c,d^ (21.50)	50 ^b^ (30.70)	35 ^b,c,d^ (21.50)	0 ^1^ (0.00)	16 ^a,c^ (9.80)	19 ^a,d^ (11.70)	<0.001
	Sometimes	133 ^a^ (28.30)	83 ^b^ (17.70)	25 ^c^ (5.30)	25 ^c^ (5.30)	96 ^b^ (20.40)	38 ^c^ (8.10)	70 ^b^ (14.90)	
	Frequently	124 ^a^ (9.80)	159 ^a,c,d^ (12.60)	204 ^b^ (16.10)	181 ^b,c^ (14.30)	191 ^b,d^ (15.10)	210 ^b^ (16.60)	195 ^b^ (15.40)	
	Always	22 ^a^ (19.60)	10 ^a,c^ (8.90)	8 ^a,c^ (7.10)	46 ^b^ (41.10)	0 ^1^ (0.00)	23 ^a^ (20.50)	3 ^c^ (2.70)	
SMOKE	Yes	274 ^a^ (15.70)	274 ^a^ (15.70)	264 ^a^ (15.20)	195 ^b^ (11.20)	184 ^b^ (10.60)	280 ^a^ (16.10)	270 ^a^ (15.50)	<0.001
	No	13 ^a^ (4.90)	13 ^a^ (4.90)	23 ^a^ (8.60)	92 ^b^ (34.30)	103 ^b^ (38.40)	7 ^a^ (2.60)	17 ^a^ (6.30)	
CH2O	Less than A L	98 ^a^ (18.20)	83 ^a,b,c^ (15.40)	80 ^a,b,c^ (14.80)	65 ^b,c^ (12.10)	92 ^a,b^ (17.10)	55 ^c^ (10.20)	66 ^a,b,c^ (12.20)	<0.001
	Between L and 2 L	138 ^a,c,e,f^ (13.80)	164 ^a,b^ (16.40)	127 ^c,d^ (12.70)	138 ^a,c,e,f^ (13.80)	97 ^d^ (9.70)	172 ^b,e^ (17.20)	164 ^b,f^ (16.40)	
	More than 2 L	51 ^a,b^ (10.90)	40 ^a^ (8.50)	80 ^b,c,e,f^ (17.00)	84 ^c,e,f^ (17.90)	98 ^c,d^ (20.90)	60 ^a,e^ (12.80)	57 ^a,f^ (12.10)	
SCC	Yes	227 ^a^ (12.60)	257 ^b^ (14.30)	276 ^c^ (15.30)	230 ^a^ (12.80)	287 ^1^(16.00)	246 ^a,b^ (13.70)	276 ^c^ (15.30)	<0.001
	No	60 ^a^ (28.60)	30 ^b^ (14.30)	11 ^c^ (5.20)	57 ^a^ (27.10)	0 ^1^ (0.00)	41 ^a,b^ (19.50)	11 ^c^ (5.20)	
FAF	I Do Not Have	83 ^a^ (9.60)	80 ^a^ (9.20)	137 ^b^ (15.80)	161 ^b,c^ (18.50)	183 ^c^ (21.10)	92 ^a^ (10.60)	132 ^b^ (15.20)	<0.001
	1 or 2 days	44 ^a^ (10.40)	97 ^b,d,e^ (22.80)	70 ^a,b,e^ (16.50)	10 ^c^ (2.40)	0 ^1^ (0.00)	130 ^d^ (30.60)	74 ^e^ (17.40)	
	2 or 4 days	137 ^a^ (24.80)	69 ^b^ (12.50)	48 ^b,c^ (8.70)	116 ^a^ (21.00)	104 ^a^ (18.80)	39 ^c^ (7.10)	39 ^c,d^ (7.10)	
	4 or 5 days	23 ^a^ (14.00)	41 ^a^ (25.00)	32 ^a^ (19.50)	0 ^1^ (0.00)	0 ^1^ (0.00)	26 ^a^ (15.90)	42 ^a^ (25.60)	
TUE	0–2 h	123 ^a,e^ (11.90)	129 ^a,b,e^ (12.50)	165 ^b,c,f^ (16.00)	173 ^c,f^ (16.80)	98 ^a^ (9.50)	193 ^c,d^ (18.70)	150 ^e,f^ (14.50)	<0.001
	3–5 h	111 ^a,c,f^ (15.90)	122 ^a^ (17.50)	72 ^b^ (10.30)	81 ^b,c^ (11.60)	189 ^d^ (27.10)	40 ^e^ (5.70)	83 ^b,f^ (11.90)	
	More than 5 h	53 ^a^ (18.90)	36 ^a^ (12.90)	50 ^a^ (17.90)	33 ^a^ (11.80)	0 ^1^ (0.00)	54 ^a^ (19.30)	54 ^a^ (19.30)	
CALC	No	0 ^1^ (0.00)	1 ^a^ (100.00)	0 ^1^ (0.00)	0 ^1^ (0.00)	0 ^1^ (0.00)	0 ^1^ (0.00)	0 ^1^ (0.00)	<0.001
	Sometimes	6 ^a^ (3.00)	18 ^a,b,d^ (9.10)	37 ^b,c,d^ (18.80)	58^c^ (29.40)	0 ^1^ (0.00)	28 ^d,e^ (14.20)	50 ^c,e^ (25.40)	
	Frequently	171 ^a,b^ (14.80)	161 ^a,b^ (13.90)	150 ^a,d^ (12.90)	166 ^a,b^ (14.30)	192 ^b^ (16.60)	195 ^b,c^ (16.80)	124 ^d^ (10.70)	
	Always	110 ^a^ (16.90)	107 ^a^ (16.40)	100 ^a^ (15.30)	63 ^b^ (9.70)	95 ^a,b^ (14.60)	64 ^b^ (9.80)	113 ^a^ (17.30)	
MTRANS	Automobile	33 ^a^ (6.30)	45 ^a,c^ (8.50)	95 ^b,d^ (18.00)	89 ^b,d^ (16.90)	91 ^b,d^ (17.20)	69 ^b,c^ (13.10)	106 ^d^ (20.10)	<0.001
	Motorbike	0 ^1^ (0.00)	4 ^a^ (14.80)	0 ^1^ (0.00)	14 ^b^ (51.90)	0 ^1^ (0.00)	9 ^a,b^ (33.30)	0 ^1^ (0.00)	
	Bike	0 ^1^ (0.00)	6 ^a^ (28.60)	9 ^a^ (42.90)	0 ^1^ (0.00)	0 ^1^ (0.00)	2 ^a^ (9.50)	4 ^a^ (19.00)	
	Public transportation	212 ^a^ (17.00)	200 ^a^ (16.10)	179 ^a,c^ (14.40)	126 ^b^ (10.10)	196 ^a^ (15.70)	181 ^a,c^ (14.50)	151 ^b,c^ (12.10)	
	Walking	42 ^a,d,e,f^ (22.30)	32 ^a,b^ (17.00)	4 ^c^ (2.10)	58 ^d^ (30.90)	0 ^1^ (0.00)	26 ^b,e^ (13.80)	26 ^b,f^ (13.80)	

Note: Values in the same row and subtable not sharing the same superscript are significantly different at *p* < 0.05 in the two-sided test of equality for column proportions; ^1^ this category was not used in comparisons because its column proportion was equal to zero or one.

**Table 3:** The hyperparameter optimization details of the machine-learning models used for obesity level estimation.

Model Name	Validation Accuracy	Hyperparameter Name	Hyperparameter Space Type	Hyperparameter Spaces	Optimal Value
LR	95.01%	C	Categorical	2^−15^, 2^−14^, 2^−13^, …, 2^13^, 2^14^, 2^15^	2^7^
		Maximum Iterations	Integer	Low = 50, High = 1000	286
RF	93.35%	Number of Estimators	Integer	Low = 50, High = 1000	527
		Maximum Depth	Integer	Low = 50, High = 1000	992
XGBoost	98.67%	Number of Estimators	Integer	Low = 50, High = 1000	998
		Maximum Depth	Integer	Low = 50, High = 1000	80
		Boosters	Categorical	‘gbtree’, ‘dart’, ‘gblinear’	gbtree
		Learning Rate	Real	Low = 10^−9^, High = 10^−1^	0.1

LR: logistic regression; RF: random forest; XGBoost: Extreme Gradient Boosting; C: The parameters are integers that instruct the model on how to handle the characteristics.

**Table 4:** Performance measures of machine-learning models with optimal hyperparameters.

Model	Accuracy	Precision	F1-Score	AUC	Recall
**LR**	**98.79%**	**99.95%**	**98.78%**	**99.99%**	**98.81%**
RF	95.57%	98.86%	95.62%	99.77%	95.58%
XGBoost	95.77%	98.25%	95.76%	99.63%	95.80%

LR: logistic regression; RF: random forest; XGBoost: Extreme Gradient Boosting; AUC: area under the curve.

**Table 5:** The hyperparameter optimization details of the machine-learning models for the feature selection phase.

Model Name	Validation Accuracy	Selected Features	Hyperparameter Name	Hyperparameter Space Type	Hyperparameter Spaces	Optimal Value
LR	99.33%	Gender, Height, Weight, History, FCVC, FAF	C	Categorical	2^−15^, 2^−14^, 2^−13^, ….., 2^13^, 2^14^, 2^15^	2^11^
			Maximum Iterations	Integer	Low = 50, High = 1000	280
			Number of Features	Integer	Low = 1, High = 12	6
RF	94.01%	Gender, Height, Weight, MTRANS	Number of Estimators	Integer	Low = 50, High = 1000	388
			Maximum Depth	Integer	Low = 50, High = 1000	53
			Number of Features	Integer	Low = 1, High = 12	4
XGBoost	94.35%	Gender, Height, Weight, History, FAF, SCC, MTRANS	Number of Estimators	Integer	Low = 50, High = 1000	980
			Maximum Depth	Integer	Low = 50, High = 1000	969
			Boosters	Categorical	‘gbtree’, ‘dart’, ‘gblinear’	‘gbtree’
			Learning Rate	Real	Low = 10^−9^, High = 10^−1^	0.002665
			Number of Features	Integer	Low = 1, High = 12	7

LR: logistic regression; RF: random forest; XGBoost: Extreme Gradient Boosting; C: The parameters are integers that instruct the model on how to handle the characteristics.

**Table 6:** Performance measures of machine-learning models with optimal hyperparameters and the selected features.

Model	Accuracy	Precision	F1-Score	AUC	Recall
**LR**	**98.99%**	**99.83%**	**98.99%**	**99.96%**	**99.01%**
RF	96.17%	98.94%	96.18%	99.76%	96.19%
XGBoost	95.77%	99.16%	95.75%	99.82%	95.80%

LR: logistic regression; RF: random forest; XGBoost: Extreme Gradient Boosting; AUC: area under the curve.

## Data Availability

The data are available for research purposes upon reasonable request to the corresponding author.
